# Size-Frequency Distributions along a Latitudinal Gradient in Middle Permian Fusulinoideans

**DOI:** 10.1371/journal.pone.0038603

**Published:** 2012-06-07

**Authors:** Yichun Zhang, Jonathan L. Payne

**Affiliations:** 1 State Key Laboratory of Palaeobiology and Stratigraphy, Nanjing Institute of Geology and Palaeontology, CAS, Nanjing, Jiangsu, China; 2 Department of Geological and Environmental Sciences, Stanford University, Stanford, California, United States of America; 3 School of Life and Environmental Sciences, Deakin University, Melbourne, Australia; Ludwig Maximilian University Munich, Germany

## Abstract

Geographic gradients in body size within and among living species are commonly used to identify controls on the long-term evolution of organism size. However, the persistence of these gradients over evolutionary time remains largely unknown because ancient biogeographic variation in organism size is poorly documented. Middle Permian fusulinoidean foraminifera are ideal for investigating the temporal persistence of geographic gradients in organism size because they were diverse and abundant along a broad range of paleo-latitudes during this interval (∼275–260 million years ago). In this study, we determined the sizes of Middle Permian fusulinoidean fossils from three different paleo-latitudinal zones in order to examine the relationship between the size of foraminifers and regional environment. We recovered the following results: keriothecal fusulinoideans are substantially larger than nonkeriothecal fusulinoideans; fusulinoideans from the equatorial zone are typically larger than those from the north and south transitional zones; neoschwagerinid specimens within a single species are generally larger in the equatorial zone than those in both transitional zones; and the nonkeriothecal fusulinoideans Staffellidae and Schubertellidae have smaller size in the north transitional zone. Fusulinoidean foraminifers differ from most other marine taxa in exhibiting larger sizes closer to the equator, contrary to Bergmann's rule. Meridional variation in seasonality, water temperature, nutrient availability, and carbonate saturation level are all likely to have favored or enabled larger sizes in equatorial regions. Temporal variation in atmospheric oxygen concentrations have been shown to account for temporal variation in fusulinoidean size during Carboniferous and Permian time, but oxygen availability appears unlikely to explain biogeographic variation in fusulinoidean sizes, because dissolved oxygen concentrations in seawater typically increase away from the equator due to declining seawater temperatures. Consequently, our findings highlight the fact that spatial gradients in organism size are not always controlled by the same factors that govern temporal trends within the same clade.

## Introduction

Body size correlates strongly with many aspects of physiology, ecology, and life history, and is therefore one of the most important attributes of any organism [1], [2]. Spatial variation in body size within and among species across large environmental gradients has been used commonly in efforts to identify the factors that most strongly influence size evolution [3]–[7]. In some cases, the factors controlling biogeographic gradients appear also to account for temporal trends (e.g. [8], [9]). In other cases, biogeographic variation in size has been used to support hypotheses regarding temporal patterns; for example, biogeographic correlation of amphipod size with oxygen availability has been interpreted as evidence in support of temporal variation in atmospheric *p*O_2_ as a control on insect size [5].

Some of the most persistent and pronounced large-scale environmental gradients are associated with latitude, which controls temperature and seasonality, among other variables. Not surprisingly, then, one of the best documented patterns of variation in organism size is the tendency for body size in endotherms to increase with increasing latitude, commonly known as Bergmann's Rule [10], [11]. This pattern has been documented in birds [12], mammals [13], and fresh-water fishes [14], although there are also many exceptions [15], [16]. Bergmann's Rule was initially interpreted to reflect the greater thermal inertia associated with larger body size [10], [17], but more recent analyses suggest other factors are likely responsible for the pattern (e.g. [18]).

Many marine animals also exhibit systematic variation in size with latitude [6], [7], [14], [19]–[29]. Many species and higher taxa show a positive association between size and latitude: fish [14], [23], *Berryteuthis anonychus* (Cephalopoda) [19], *Tegula funebralis* and *Troschelia berniciensis* (gastropods) [22], [25], and terebratulid brachiopods [28]. By contrast, Late Cenozoic planktonic foraminifera show larger size toward equatorial regions [30], [31]. There are also taxa that exhibit no size change across latitude, such as echinoids and bivalves [6], [24], [26].

The extent to which Bergmann's rule has been temporally persistent is unclear because there have been few analyses of body size gradients using fossil material. Adding a temporal dimension to the analysis of biogeographic variation in organism size has the potential to shed additional insight on underlying controls. Whereas the basic environmental gradients on the planet controlled by latitude-dependent solar insolation have remained relatively constant through geological time, the ecological structure of global ecosystems has changed substantially (e.g. [32]–[35]). Consequently, documentation of ancient biogeographic patterns of body size variation can provide additional insight into the extent to which these patterns are controlled by aspects of the physical environment versus ecological interactions.

**Figure 1:**
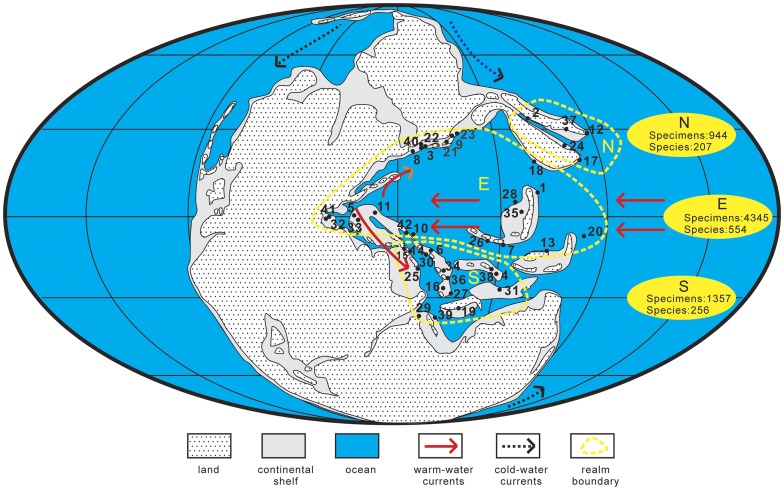
Middle Permian paleogeographic map showing three realms and blocks containing fusulinoideans in the analysis (base map modified after [**68**]). Abbreviations/key: N, north transitional zone; E, equatorial zone; S, south transitional zone; 1, Akiyoshi Terrane; 2, Altaid Belt; 3, Armenia; 4, Baoshan Block; 5, Carnic Alps; 6, Central Iran; 7, Changning-Menglian Belt; 8, Crimea; 9, Darvaz; 10, exotic Karakaya complex in Turkey; 11, Greece; 12, Hida Gaien Belt; 13, Indochina Block; 14, Iraq; 15, Israel; 16, Karakorum; 17, Kitakami Terrane; 18, Kunlun-Qadam Block; 19, Lhasa Block; 20, exotic blocks in New Zealand; 21, north Afghanistan; 22, north Caucasus; 23, north Pamir; 24, northern margin of North China Block; 25, Oman; 26, Qamdo Block; 27, Qiangtang Block; 28, Qinling Belt; 29, Salt Range; 30, Sanandaj-Sirjan zone of Iran; 31, Sibumasu Block; 32, Sicily; 33, Slovenia; 34, south Afghanistan; 35, South China; 36, south Pamir; 37, South Primorye; 38, Tengchong Block; 39, Tethys Himalaya; 40, Transcaucasia; 41, Tunisia; 42, Turkey.

Fusulinoidean foraminifera present an ideal opportunity to begin to examine ancient patterns of biogeographic variation in organism size. They were abundant and diverse during Late Paleozoic time and span over six orders of magnitude in test (i.e. shell) volume, from 0.001 mm^3^ to more than 1000 mm^3^. In addition, the time during which they evolved was marked by drastic climatic change, from ice-house conditions during the Late Carboniferous and Early Permian to greenhouse conditions during the latest Permian [36]. The climatic transition and active tectonic movement led to the development of distinct paleobiogeographic zones within the Tethys during the Middle Permian: an equatorial zone and two temperate zones in the southern and northern hemispheres, reflected in the biogeography of brachiopods [37]–[39], corals [40], [41] and fusulinoideans [42]–[44]. Fusulinoideans, in particular, have not been discovered in high paleo-latitude deposits so far, such as those of cold-water India and Australia [42], [43]. Thus, there is clear biogeographic evidence of environmental limitation of fusulinoidean occurrence patterns. The extent to which those same factors influenced fusulinoidean sizes has not been examined previously.

Fusulinoideans also exhibit substantial variation in mean and maxium size of species across time, increasing during the Late Carboniferous and Early Permian, and then declining during the later part of the Middle Permian and, especially, the Late Permian [45]. Secular variation in fusulinoidean size is best explained by coeval variation in atmospheric oxygen levels, which also impact oxygen availability to shallow-marine, epifaunal organisms such as fusulinoideans [45]. The extent to which variation in oxygen availability may also account for spatial variation in fusulinoidean size remains unstudied.

In this study, we quantify the body sizes of Middle Permian fusulinoidean fossils from three different paleo-latitude zones within the Tethys Ocean. We use these results to investigate controls on biogeographic variation in fusulinoidean size and discuss the relationship between controls on spatial and temporal variation in fusulinoidean size.

## Methods

### Material

We compiled size data for Middle Permian fusulinoideans from 194 monographs illustrating Middle Permian fusulinoideans from the Tethyan region, together with one author's [Z.Y.] unpublished data from the south transitional zone (Fig. 1). The length and diameter of each fusulinoidean specimen was either compiled from tables or was measured from figures if no size data were provided explicitly in the monograph. Volumes were calculated by assuming that fusulinoideans are approximately three-dimensional ellipsoids: V = 4/3·π·*r* ^2^·*l* where *r* represents the radius and *l* the half-length.

**Figure 2:**
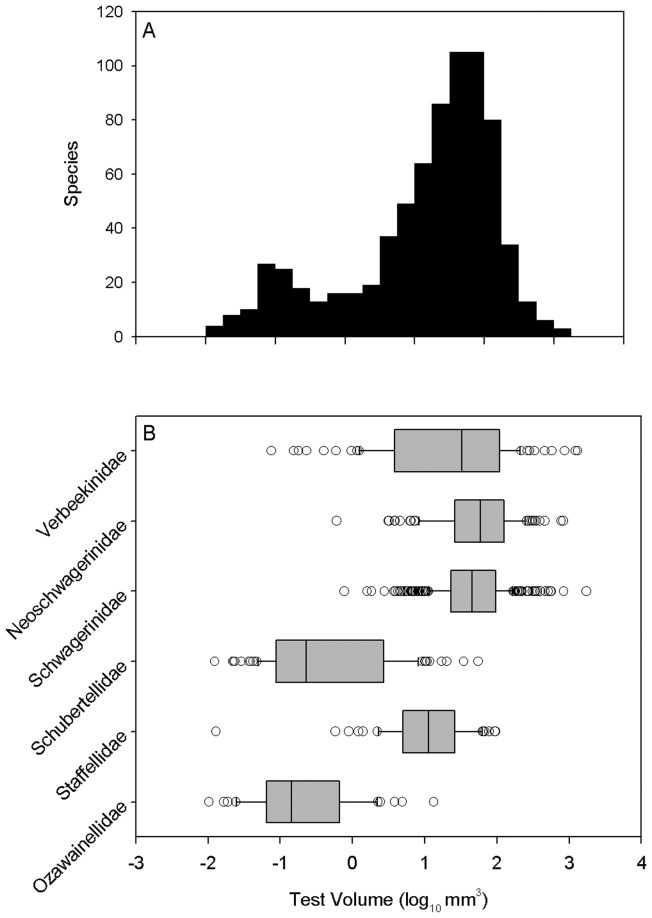
Size distribution of Middle Permian fusulinoidean species, showing bimodal, left-skewed distribution resulting from differences in size and diversity among families. (A) All species. (B) Size distributions of families. Boxes present interquartile range, with median indicated by a black line. Whiskers indicate 5^th^ and 95^th^ percentiles. Species beyond the 5^th^ and 95^th^ percentiles are indicated individually.

We standardized taxonomic assignments to the greatest extent possible prior to analysis. The revised database includes 738 species referable to 74 genera and 6 families (Neoschwagerinidae, Verbeekinidae, Schwagerinidae, Staffellidae, Schubertellidae and Ozawainellidae) (Table S1).

### Statistical Analysis

For all analyses and associated figures, we used only the largest specimen to represent each species, or the largest specimen of the species within a given region to represent that species within the region. This approach minimizes the influence of juvenile or incompletely preserved specimens and has been used extensively in the study of size evolution within marine organisms that exhibit indeterminate growth [46]–[48]. The use of specimens illustrated for taxonomic purposes in monographs further reduces the influence of poorly oriented or substantially incomplete specimens. Although indeterminate growth complicates the measurement of a representative size, we note that indeterminately growing organisms typically exhibit a reduction in growth rate over time (in terms of linear dimensions). This slowing is even more pronounced in the logarithmic space used for the study of body size evolution. Moreover, indeterminately growing organisms do not live for arbitrarily long periods of time. Thus, even if these organisms grow throughout their lifetimes, they do not grow to arbitrarily large sizes; this fact is implicit in the use of body size as a taxon character for many species of indeterminately growing organisms, including foraminifera. All statistical analyses were performed using SAS (version 9.2).

**Figure 3:**
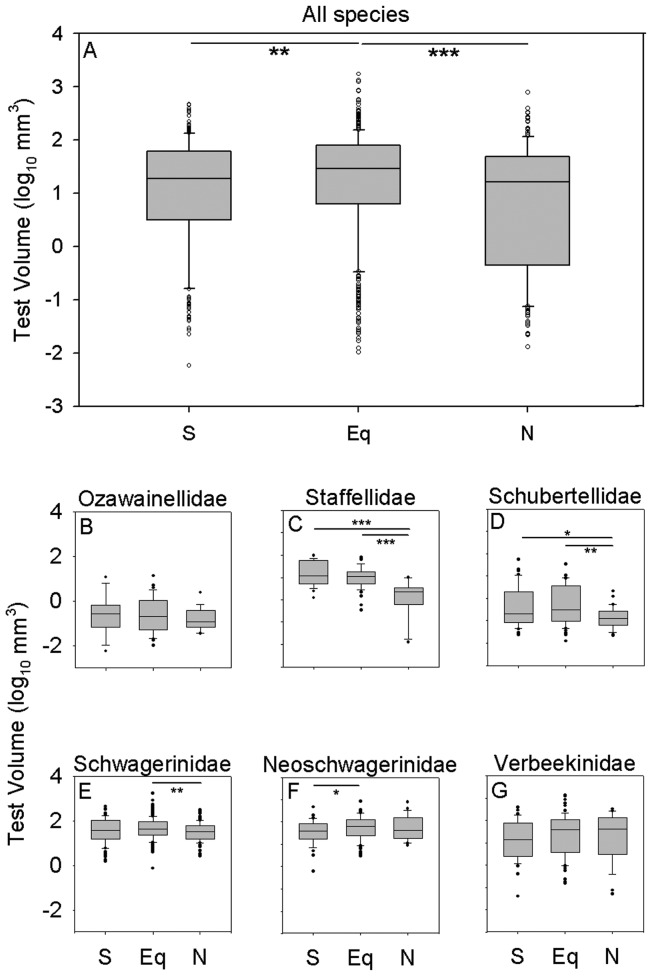
Comparison of size distributions of species between the equatorial region and the north and south transitional zones. Overall, equatorial species are significantly larger than species from the transitional zones, but the direction and magnitude of size differences between regions varies among families. (A) All species. (B) Ozawainellidae. (C) Staffellidae. (D) Schubertellidae. (E) Schwagerinidae. (F) Neoschwagerinidae. (G) Verbeekinidae. Boxes and whiskers as in Figure 2. * *p*<0.05; ** *p*<0.01; *** *p*<0.001. Significance levels of all comparisons are presented in Table 1.

## Results

Middle Permian fusulinoideans span more than five orders of magnitude in test (i.e. shell) volume, from 0.01 mm^3^ to more than 1000 mm^3^ (Fig. 2A). The overall size distribution among species is left-skewed and bi-modal, with a major mode at large size (10 to 100 mm^3^) and a minor mode at small size (0.1 mm^3^). The complex size distribution results from substantial differences in size distribution species among fusulinoidean families. Species in the families Verbeekinidae, Neoschwagerinidae and Schwagerinidae are substantially larger than species in the families Schubertellidae, Staffellidae and Ozawainellidae (Fig. 2B). The former families are more diverse and define the major size mode, whereas the less diverse schubertellids and ozaiwanellids define the smaller size mode.

**Figure 4:**
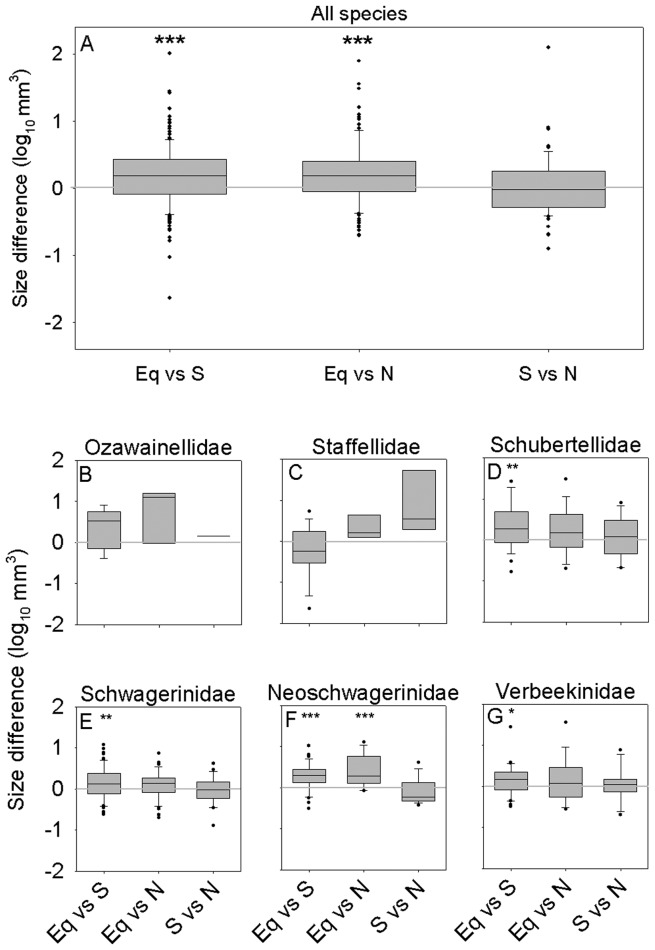
Intraspecific size differences between regions, illustrated using the largest specimen for each species in each region. The graph illustrates the tendency for the largest equatorial specimen of a given species to be larger than the largest conspecific specimen from the transitional zones. (A) All species. (B) Ozawainellidae. (C) Staffellidae. (D) Schubertellidae. (E) Schwagerinidae. (F) Neoschwagerinidae. (G) Verbeekinidae. Boxes and whiskers as in Figure 2. * *p*<0.05; ** *p*<0.01; *** *p*<0.001. Significance levels of all comparisons are presented in Table 2.

**Figure 5:**
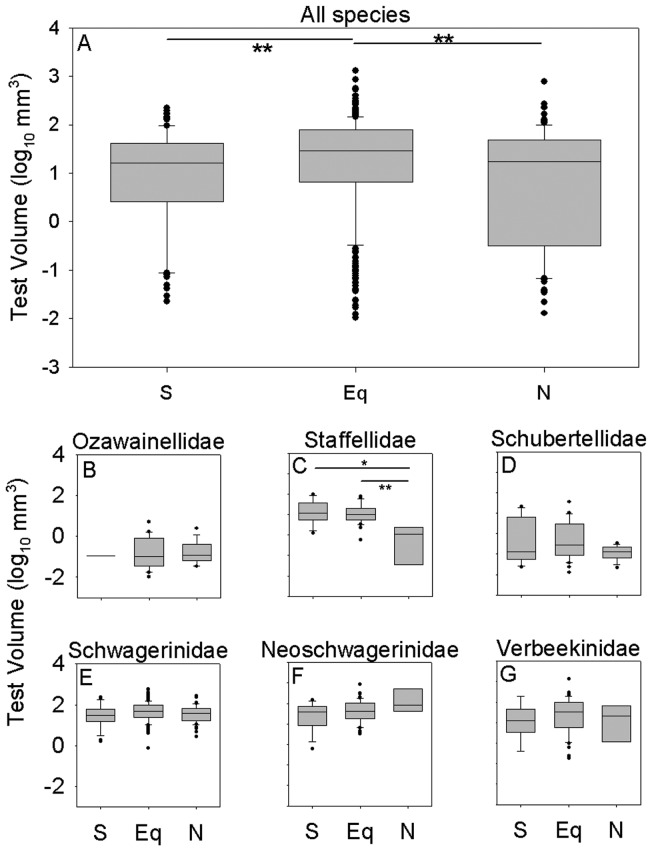
Size distributions of species endemic to indicated regions, using the largest specimen for each species in each region. (A) All species. (B) Ozawainellidae. (C) Staffellidae. (D) Schubertellidae. (E) Schwagerinidae. (F) Neoschwagerinidae. (G) Verbeekinidae. Boxes and whiskers as in Figure 2. * *p*<0.05; ** *p*<0.01; *** *p*<0.001. Significance levels of all comparisons are presented in Table 3.

**Table 1:** Results of Wilcoxon two-sample tests (t approximation) comparing median values of size distributions between regions for all fusulinoidean species and for species within individual families.

	Eq. vs. S.	Eq. vs. N.	S. vs. N.
All species	N_eq_ = 554 N_s_ = 256 U = 95397 **p = 0.007**	N_eq_ = 554 N_n_ = 207 U = 68656.5 **p = 0.0002**	N_n_ = 207 N_s_ = 256 U = 46278 p = 0.22
Neoschwagerinidae	N_eq_ = 100 N_s_ = 49 U = 3180 **p = 0.048**	N_eq_ = 100 N_n_ = 23 U = 1403 p = 0.88	N_n_ = 23 N_s_ = 49 U = 944 p = 0.21
Ozawainellidae	N_eq_ = 33 N_s_ = 12 U = 278 p = 0.97	N_eq_ = 33 N_n_ = 18 U = 430 p = 0.46	N_n_ = 18 N_s_ = 12 U = 209 p = 0.35
Schubertellidae	N_eq_ = 66 N_s_ = 44 U = 2321 p = 0.46	N_eq_ = 66 N_n_ = 36 U = 1379 **p = 0.001**	N_n_ = 36 N_s_ = 44 U = 1228 **p = 0.03**
Schwagerinidae	N_eq_ = 230 N_s_ = 85 U = 12581 p = 0.24	N_eq_ = 230 N_n_ = 95 U = 13259 **p = 0.004**	N_n_ = 95 N_s_ = 85 U = 8052 p = 0.31
Staffellidae	N_eq_ = 46 N_s_ = 27 U = 1076 p = 0.39	N_eq_ = 46 N_n_ = 10 U = 113 **p = 0.0006**	N_n_ = 10 N_s_ = 27 U = 83 **p = 0.0008**
Verbeekinidae	N_eq_ = 79 N_s_ = 39 U = 2103 p = 0.22	N_eq_ = 79 N_n_ = 25 U = 1325 p = 0.93	N_n_ = 25 N_s_ = 39 U = 885 p = 0.33

Corresponding size distributions are illustrated in Figure 3. Bold type indicates statistical significance at α = 0.05. Abbreviations: Eq, equatorial zone; N, north transitional zone; S, south transitional zone.

There is substantial evidence for variation in the overall size distribution of fusulinoidean species as a function of latitude (Fig. 3A). The mean values of size distribution for equatorial fusulinoidean species are significantly larger than those for the north and south transitional zones (Mann Whitney U-test: equatorial versus north transitional zone, two-tailed *p* = 0.0002; equatorial versus south transitional zone, two-tailed *p* = 0.007; see also Table 1). A bootstrap resampling analysis (10,000 replicates) of species from the equatorial region shows that the differences in maximum size between the equatorial region and transitional zones are also larger than can be explained simply by differences in diversity (bootstrap resampling using 10,000 replicates: equatorial versus south, *p* = 0.007; equatorial versus north, *p* = 0.10) but the second-largest species from the north is substantially smaller (2.89 versus 2.59 log mm^3^). Median sizes across species are statistically indistinguishable between the north and south transitional zones (Mann Whitney U-test: *p* = 0.22). These size differences result from both within- and among-species differences in size. The largest equatorial representative of a species is on average significantly larger than the largest conspecific specimen from either transitional zone (Fig. 4A; two-tailed t-test: equatorial versus north transitional zone, *p*<0.0001; equatorial versus south transitional zone, *p*<0.0001), whereas there is no tendency for the largest representative of a given species from the north transitional zone to be either larger or smaller than the largest conspecific specimen from the south transitional zone (Fig. 4A; two-tailed t-test: *p* = 0.43). Species endemic to the equatorial zone are also larger, on average, than species endemic to either transitional zone (Fig. 5A; two-tailed Mann-Whitney test: equatorial versus north transitional zone, *p* = 0.002; equatorial versus south transitional zone, *p* = 0.006) whereas the median sizes of endemics from the two transitional zones are statistically indistinguishable from one another (two-tailed Mann-Whitney test: *p* = 0.83).

**Table 2:** Results of t-tests comparing mean values of within-species size differences between regions to a null hypothesis of no size difference between regions.

	Eq. vs. S.	Eq. vs. N.	S. vs. N.
All species	N = 170 t = 5.23 **p<0.0001**	N = 103 t = 5.32 **p<0.0001**	N = 61 t = 0.80 p = 0.43
Neoschwagerinidae	N = 34 t = 4.79 **p<0.0001**	N = 18 t = 4.46 **p = 0.0003**	N = 16 t = −1.13 p = 0.28
Ozawainellidae	N = 9 t = 2.09 p = 0.07	N = 3 t = 1.94 p = 0.19	N = 2 t = 0.51 p = 0.70
Schubertellidae	N = 25 t = 2.87 **p = 0.008**	N = 19 t = 1.77 p = 0.09	N = 11 t = 0.33 p = 0.75
Schwagerinidae	N = 58 t = 3.21 **p = 0.002**	N = 39 t = 3.07 **p = 0.004**	N = 17 t = −0.25 p = 0.80
Staffellidae	N = 14 t = −1.59 p = 0.14	N = 6 t = 1.42 p = 0.21	N = 4 t = 2.02 p = 0.14
Verbeekinidae	N = 30 t = 2.17 **p = 0.04**	N = 18 t = 1.28 p = 0.22	N = 11 t = 0.33 p = 0.75

Sample sizes represent that number of species that occur in both regions. Corresponding distributions of intraspecific size differences are illustrated in Figure 4. Bold type indicates statistical significance at α = 0.05. Abbreviations: Eq, equatorial zone; N, north transitional zone; S, south transitional zone.

**Table 3:** Results of Wilcoxon two-sample tests (t approximation) comparing median values of size distributions between regions for species endemic to a single region.

	Eq. vs. S.	Eq. vs. N.	S. vs. N.
All species	N_eq_ = 336 N_s_ = 80 U = 13999 **p = 0.006**	N_eq_ = 338 N_n_ = 98 U = 17979.5 **p = 0.002**	N_n_ = 98 N_s_ = 80 U = 7236 p = 0.83
Neoschwagerinidae	N_eq_ = 63 N_s_ = 14 U = 467 p = 0.30	N_eq_ = 63 N_n_ = 4 U = 191****p = 0.15	N_n_ = 4 N_s_ = 14 U = 56 p = 0.08
Ozawainellidae	N_eq_ = 22 N_s_ = 2 U = 26 p = 0.96	N_eq_ = 22 N_n_ = 14 U = 272 p = 0.69	N_n_ = 14 N_s_ = 2 U = 16 p = 0.94
Schubertellidae	N_eq_ = 30 N_s_ = 16 U = 354 p = 0.62	N_eq_ = 30 N_n_ = 14 U = 237 p = 0.06	N_n_ = 14 N_s_ = 16 U = 199 p = 0.47
Schwagerinidae	N_eq_ = 149 N_s_ = 26 U = 1862 p = 0.08	N_eq_ = 149 N_n_ = 55 U = 4995 p = 0.09	N_n_ = 55 N_s_ = 26 U = 999 p = 0.50
Staffellidae	N_eq_ = 30 N_s_ = 13 U = 299 p = 0.74	N_eq_ = 30 N_n_ = 4 U = 14 p = 0.006	N_n_ = 4 N_s_ = 13 U = 13 **p = 0.02**
Verbeekinidae	N_eq_ = 42 N_s_ = 9 U = 195 p = 0.35	N_eq_ = 42 N_n_ = 7 U = 139 p = 0.32	N_n_ = 7 N_s_ = 9 U = 59 p = 1.00

Sample sizes represent the numbers of species endemic to the indicated regions. Corresponding size distributions are illustrated in Figure 5. Bold type indicates statistical significance at α = 0.05. Abbreviations: Eq, equatorial zone; N, north transitional zone; S, south transitional zone.

Within this broader pattern of size decrease from the equatorial region to the transitional zones, biogeographic patterns of size variation differ among families (Fig. 3B–G). The schwagerinids and neoschwagerinids, which show the largest overall sizes, consistently exhibit larger median sizes across species in the equatorial zone and smaller median sizes across species in the transitional zones, although only some of these differences are statistically significant (Fig. 3B–G; Table 1). In contrast, families with smaller overall mean size exhibit a variety of patterns. Schubertellids and staffellids exhibit significantly smaller sizes in the north transitional zone than in either of the other regions, whereas ozawainellids and verbeekinids exhibit no statistically significant variation in size across regions.

Regional size gradients within families result more from size gradients within species (Fig. 4B–G; Table 2) than from size differences between endemics of different regions (Fig. 5B–G; Table 3). Schubertellids, schwagerinids, neoschwagerinids, and verbeekinids all show evidence of intraspecific size gradients. In all cases where significant differences occur, the largest equatorial representatives tend to be larger than the largest conspecific specimens from the transitional zones. In contrast, only staffellids show evidence for significant size differences between species endemic to different zones, with species endemic to the north transitional zone being smaller than those endemic to the equatorial and south transitional zones (Fig. 5D).

## Discussion

Our results show that equatorial fusulinoideans exhibit larger median and maximum sizes than their counterparts from the north and south transitional zones, and this is largely controlled by gradients within species. A number of factors have been proposed to explain size gradients in the marine realm, including temperature [8], [9], [22], dissolved oxygen concentrations [4], [5], surface-water stratification intensity [30], [31], food availability [26], mortality [24], and competition [26], [28], [49]. Nutrient availability, dissolved oxygen availability, and the presence of photosymbionts have all been proposed as controls on foraminiferal size [50]–[53].

Given the broad scope of this study, local variations in habitat, water depth, and salinity are unlikely to account for the overall pattern of size decrease away from the equatorial region.

Several other factors can be ruled out because the predicted sense of size variation is opposite to that observed. Oxygen solubility is greater in colder waters; consequently, an oxygen control on size would tend to produce larger sizes at higher latitudes, consistent with the polar gigantism exhibited by living pycnogonids, isopods, nemerteans, and amphipods [4], [5]. Similarly, metabolic rates tend to be lower at colder temperatures, further reducing the constraints of oxygen availability on organism size [54]. The biogeographic patterns of size variation in fusulinoideans thus contrast with evidence for oxygen availability as an important control on maximum foraminiferal size in the deep sea since the Cretaceous [55] as well as on fusulinoideans themselves through the Carboniferous and Permian [45].

Algal symbiosis is widely hypothesized as a driver of gigantism in fusulinoideans and other larger foraminifera. Algal symbiosis provides energy to host cells and thereby enhances calcification [56]. Symbiont-bearing foraminifers are capable of occupying low-nutrient environments and generating several orders of magnitude energetic advantage over non-symbiotic competitors sharing the same environments [50], [56], [57]. Evidence for algal symbiosis in fusulinoideans is primarily indirect. Lee and Hallock (1987) reported the remains of an endosymbiotic alga in the shell of the fusulinoidean *Pseudoschwagerina montanensis*, but the interpretation of widespread symbiosis in fusulinoideans has been based primarily on analogy to large, internally complex, symbiont-bearing modern species [58]–[63]. Based upon this comparison, the comb-like keriothecal wall structure in Neoschwagerinidae, Schwagerinidae and Verbeekinidae is widely regarded as an adaptation to house algal symbionts [58], [62], [63]. If correct, this interpretation would explain why species in the keriothecal-walled families Verbeekidae, Neoschwagerinidae, Schwagerinidae are much larger than members of the Schubertellidae, Staffellidae and Ozawainellidae (Fig. 2B).

Seasonality is another potentially important factor affecting the growth of foraminifers. The length of the growing season in the tropics is several times longer than in temperate waters [59]. If seasonality is accompanied by episodes of increased risk of mortality, minimal seasonality and low mortality in the tropics may explain selection for larger size in this region [50].

A final factor that may influence the spatial gradient in fusulinoidean sizes is the calcium carbonate saturation level of seawater. This is typically highest in warm, well-stratified equatorial waters and diminishes toward the poles due to the temperature dependence of carbonate mineral solubility and greater mixing of CO_2_-charged deep waters in areas of reduced density contrast between surface and deep waters [64]. Because the carbonate content of fusulinoidean tests per unit volume typically increases with size, the greater relative energetic demands of secreting the test at higher latitudes under lower levels of calcium carbonate saturation may explain at least part of the size gradient between equatorial and transitional zones.

The primary biogeographic size gradient in Middle Permian fusulinoideans is between the equatorial region and the transitional zones, but differences in size distributions between the north and south transitional zones may also be related to differences in water mass characteristics. The south transitional zone differs from the equatorial zone in its smaller keriothecal fusulinoideans such as Neoschwagerinidae and Schwagerinidae. However, the nonkeriothecal fusulinoideans Staffellidae and Schubertellidae in the north transitional zone also show smaller size by contrast with the equatorial zone. The north transitional zone contains faunas of mixed tropical and cool temperate affinities: brachiopods, bivalves, fusulinoideans, ammonoids, bryozoans and conodonts [37], [65]. The mixed fauna has been interpreted as either the result of northward drift of Sino-Korean platform or the flowing down of cold-water currents in the Arctic Ocean along the northeastern margin of Siberian platform (Fig. 1) [39], [66]. By contrast, with the northward drift of the Cimmerian Continent and climatic amelioration after the Late Paleozoic Ice Age, the cold-water fauna appears to have vanished after the Wordian [39]. Consequently, it is clear that, during the Middle Permian, the north transitional zone was more affected by cold water than the south transitional zone. The circulation of cold currents in the north transitional zone may be responsible for its overall smaller fusulinoidean size distribution (Fig. 2). In particular, the nonkeriothecal Staffellidae and Schubertellidae in the north transitional zone also exhibit smaller sizes when compared to the equatorial zone, in addition to Neoschwagerinidae and Schwagerinidae (Fig. 2, 3), which differs from the condition for the south transitional zone.

The complexity of the size-latitude relationship across families of fusulinoideans observed in this study is consistent with differences among previously studied marine animals. Many taxa exhibit trends toward larger size at higher latitudes (Bergmann's Rule), including some species of gastropod [19], [22], [25], crustaceans [67], crabs [20], and bryozoans [21]. However, other taxa, such as urchins and bivalves, show no latitudinal size trend [6], [24]. To our knowledge, the only documented case similar to our present study occurs in Late Cenozoic planktonic foraminifera, which also show a trend toward larger size in equatorial areas [30], [31]. These complexities suggest that there are numerous factors that contribute to biogeographic variation in body size and that the relative importance of these factors varies as a function of clade-level ecology and physiology.

In many cases, biogeographic variation is used to verify hypothesized causes of temporal change in organismal characters. For example, the consistency of spatial and temporal correlation between temperature and ostracod size strongly suggests that the same factors that produce spatial variation in the sizes of living deep-sea ostracods can account for trends toward larger size through the Cenozoic [8], [9]. Similarly, demonstration of biogeographic variation in size with respect to oxygen availability has been used to support the hypothesis that atmospheric hyperoxia was the primary driver of late Paleozoic insect gigantism (e.g. [5]). In contrast, the temporal evolution of fusulinoidean size appears to be tightly coupled to variation in atmospheric oxygen concentrations [45], whereas observed spatial variation trends are opposite in direction to predictions based upon a control by oxygen availability. These observations are not mutually incompatible: meridional gradients may have exerted strong but temporally invariant pressures on fusulinoidean size while atmospheric oxygen levels may have exerted weaker but temporally variable influences on size. Payne et al. [45] found that the mean size across fusulinoidean species increased approximately 0.25 log mm^3^ units for each percent change in atmospheric *p*O_2_. Based upon spatial variation in dissolved oxygen in the modern surface ocean, this effect should product an approximate 0.25 log mm^3^ increase in mean size from the equatorial to transitional zones, similar in magnitude, but opposite in direction, to the effect observed in this study (Fig. 3). Therefore, spatial variation in carbonate saturation, seasonality, or other factors must have imposed sufficiently strong selective pressures on test size to offset a substantial counteracting pressure from oxygen availability.

### Conclusions

Middle Permian fusulinoidean foraminifers of the Tethys exhibit pronounced biogeographic variation in test size. In particular, families containing the largest species and those with the most pronounced morphological features indicative of algal symbionts show a pronounced trend of size decrease away from the equatorial region. Much of this size decrease occurred through within-species size trends. Contrary to evidence that oxygen availability was a primary control on temporal trends in fusulinoidean sizes, our analysis shows that meridional variation in seasonality, water temperature, nutrient content, and carbonate saturation level were the most likely factors influencing biogeographic variation in size. Differences in the pattern of biogeographic size variation among marine taxa indicate considerable variation in the identities of the most important controls on size across taxa. Moreover, our findings demonstrate that the controls on temporal and spatial variation in size may differ within a single clade.

## Supporting Information

Table S1 **The Middle Permian fusulinoidean database used in the analysis.**
(XLSX)
